# Tuberculosis Phenotypic and Genotypic Drug Susceptibility Testing and Immunodiagnostics: A Review

**DOI:** 10.3389/fimmu.2022.870768

**Published:** 2022-07-07

**Authors:** Kizil A. Yusoof, Juan Ignacio García, Alyssa Schami, Andreu Garcia-Vilanova, Holden V. Kelley, Shu-Hua Wang, Adrian Rendon, Blanca I. Restrepo, Marcel Yotebieng, Jordi B. Torrelles

**Affiliations:** ^1^ Graduate School of Biomedical Sciences, University of Texas Health San Antonio, San Antonio, TX, United States; ^2^ Population Health Program, Tuberculosis Group, Texas Biomedical Research Institute, San Antonio, TX, United States; ^3^ Department of Internal Medicine, Division of Infectious Diseases, College of Medicine and Global One Health Initiative, The Ohio State University, Columbus, OH, United States; ^4^ Centro de Investigación, Prevención y Tratamiento de Infecciones Respiratorias (CIPTIR), Hospital Universitario de Monterrey Universidad Autónoma de Nuevo León (UANL), Monterrey, Mexico; ^5^ School of Public Health, University of Texas Health Science Center at Houston, Brownsville, TX, United States; ^6^ School of Medicine, South Texas Diabetes and Obesity Institute, University of Texas Rio Grande Valley, Edinburg, TX, United States; ^7^ Division of General Internal Medicine, Department of Medicine, Albert Einstein College of Medicine, New York City, NY, United States

**Keywords:** TB diagnostics, multi-drug resistance, anti-TB drug regimens, active TB, point of care (POC)

## Abstract

Tuberculosis (TB), considered an ancient disease, is still killing one person every 21 seconds. Diagnosis of *Mycobacterium tuberculosis (M.tb)* still has many challenges, especially in low and middle-income countries with high burden disease rates. Over the last two decades, the amount of drug-resistant (DR)-TB cases has been increasing, from mono-resistant (mainly for isoniazid or rifampicin resistance) to extremely drug resistant TB. DR-TB is problematic to diagnose and treat, and thus, needs more resources to manage it. Together with+ TB clinical symptoms, phenotypic and genotypic diagnosis of TB includes a series of tests that can be used on different specimens to determine if a person has TB, as well as if the *M.tb* strain+ causing the disease is drug susceptible or resistant. Here, we review and discuss advantages and disadvantages of phenotypic *vs*. genotypic drug susceptibility testing for DR-TB, advances in TB immunodiagnostics, and propose a call to improve deployable and low-cost TB diagnostic tests to control the DR-TB burden, especially in light of the increase of the global burden of bacterial antimicrobial resistance, and the potentially long term impact of the coronavirus disease 2019 (COVID-19) disruption on TB programs.

## Introduction

The World Health Organization (WHO) estimates that over the next 35 years, without proper surveillance and diagnosis, approximately 75 million people will suffer from drug resistant (DR)-tuberculosis (TB), costing the global economy $16.7 trillion dollars (1, 2). These numbers may fall short due to the projected negative impact of the coronavirus disease 2019 (COVID-19) pandemic on TB control. For decades, the WHO has relied on a simplified and pragmatic approach offering standardized drug regimens to everyone to treat TB. However, the control and management of the rising burden of DR-TB requires universal access to drug susceptibility testing (DST) and individualized treatment approaches. Currently, in high TB burden countries, there are limited diagnostic options to test for DR-TB. Tests such as the BACTEC™ Mycobacteria Growth Indicator Tubes (MGIT), Xpert or Xpert Ultra ^®^ MTB/RIF, Truenat MTB/MTB Plus/MTB-RIF DX, and Line Probe Assays (LPA), although provide results for DR-TB, are costly and require complex equipment, laboratory infrastructures, biosafety needs, and training for lab technicians. These facts limit their deployment in point of care (POC) settings in low resource communities, where DR-TB cases are rising, specially due to the impact of the COVID-19 pandemic limiting DR-TB testing and treatment (lack of drug supplies), and dedicated personnel (3). Thus, there is a need to develop improved POC diagnostic tests for prompt DR-TB diagnosis and treatment monitoring.

Despite the substantial progress in overall TB control that resulted in a 47% decrease in TB mortality between 1990 and 2015, TB still remains as one of the single infectious diseases associated with high mortality (4). The emergence of several profiles of DR-TB over the past 30 years has further complicated the use of standardized drug treatment regimens. Even pre-COVID-19, estimates indicated that DR-TB will be responsible for more deaths due to antimicrobial resistance than any other single pathogen (5).

Halting the burden of DR-TB will require access to high quality drug susceptibility testing to inform and guide individualized regimens for every TB patient, particularly in high-burden low resource settings. Here, we review the current diagnostic landscape for DR-TB including those in development to assess their suitability and sustainability for DR-TB control based on individualized regimens. Importantly, we explore the possibility of applying known serological test for active TB, as well as to determine if a given patient is responding to treatment. The need for specific *M.tb* and/or host biomarkers to differentiate drug-susceptible (DS) and DR-TB with a simple POC test is also discussed.

## Updated DR-TB Definitions

Since the early 1990s, TB prevention and care have been complicated by the growing global burden of rifampicin-resistant (RR) and multidrug-resistant (MDR)-TB. MDR-TB is defined as infection with *M.tb* resistant to at least the first line drugs isoniazid (INH) and rifampicin (RIF) (6). Recently, a WHO expert consultation meeting updated the definitions for extensively-drug resistant (XDR-TB) (7); since January 2022, pre-XDR-TB is defined as TB caused by *M.tb* strains that fulfill the definition of MDR/RR-TB and that are also resistant to any fluoroquinolone, and XDR-TB is defined as TB caused by *M.tb* strains that fulfill the definition of MDR/RR-TB and that are also resistant to any fluoroquinolone and at least one additional Group A drug. The Group A drugs are currently levofloxacin or moxifloxacin, bedaquiline and linezolid; therefore, XDR-TB is MDR/RR-TB that is resistant to a fluoroquinolone and either bedaquiline or linezolid, or both (7). Further, the terms, extremely-drug-resistant TB (XXDR-TB) and total-drug-resistant TB (TDR-TB) were proposed by specific studies to describe the cohort of patients resistant to all tested anti-TB drugs (8–11). However, these terms are not recognized by the WHO as DST is technically challenging and cannot be thoroughly tested since current drug effectiveness against TDR strains are not extensively reported (10). XXDR-TB refers to strains that are resistant to all first and second line anti-TB drugs (8). XXDR resistance, although difficult to treat, is distinct from TDR-TB. TDR-TB is reserved for *M.tb* strains that demonstrate resistance to all available first- and second-line anti-TB drugs, including drugs in the discovery pipeline (9). This drug resistance is thought to be attributed to bacterial chromosomal mutations, inadequate treatments, lengthy drug regimens, patient non-compliance, and presence of other comorbidities, among other reasons (12, 13).

In the last 15 years, while the numbers of deaths due to DS- and DR-TB are estimated to have declined by 20.6% and 28.9%, respectively, deaths due to pre-XDR/XDR-TB have significantly increased by 67.6% (14). Indeed, cases of natural polymorphisms in *M.tb* conferring resistance to the newest anti-TB drug delamanid in drug-naïve patients are reported in countries where this drug had not yet been introduced (15). Similarly, discordant results between laboratories testing the same patient’s sample, different patient’s samples, and/or between genotypic and phenotypic testing of the same sample, add a layer of complexity to the already difficult task of identifying and properly classifying DR-TB. In this context, in a recent study looking at mortality among people with TB living in various settings across the globe, researchers compared phenotypic or genotypic DS-TB test results obtained from both local and a Switzerland reference laboratories testing identical samples (16). Discordant results were found between local and reference laboratories in about 20% of the samples assessed. Mortality ranged from 6% in people with pan-susceptible TB treated according to WHO guidelines, to 57% in people with DR-TB who went under-wrong treatment because of the discordance between both the local and reference laboratories (16). Interestingly, people with INH monoresistant TB, the most common form of DR-TB globally, had a higher mortality rate compared to MDR-TB patients (16). Thus, in order to reduce TB mortality among DR-TB patients timely access to accurate DST is essential for every TB patient to inform and guide the therapeutic decision.

## Current Landscape for Universal DST

### Phenotypic DST

WHO recommends rapid diagnostics and universal DST (at least RIF resistance testing) for all people experiencing clinical symptoms of active TB, although the current available tools make this goal impossible (17, 18). There are a number of genotypic and phenotypic DST diagnostic tools available for DR-TB diagnosis; however, their optimal use and their results interpretation are critical for a timely and accurate DR-TB diagnosis to enable an effective patient treatment and care (19).

Among the phenotypic DST used for DR-TB diagnosis, culture, both solid and liquid, is the most commonly used method. Thus, a confirmed *M.tb* specimen is further cultured in solid or liquid media containing the critical concentration of a given anti-TB drug. Lack of *M.tb* growth indicates susceptibility to a given drug, while *M.tb* growth indicates resistance. Culture also provides information on the critical and the minimum inhibitory concentrations (MIC), where MIC is the lowest anti-TB drug concentration capable of inhibiting the growth of a *M.tb* strain.

Phenotypic DST in solid medium is standardized and the most frequently used form of DST in mid and low-income areas endemic for TB (**Table 1**). Löwenstein–Jensen (LJ) slants is the most widely used medium followed by Middlebrook (M) 7H10 or 7H11 agar (20). *M.tb* growth is visualized by the typical rough colonies and cording formations. The indirect proportion method is the most commonly used for solid medium DST using a standardized and two 10-fold diluted dilutions of the inoculum with the anti-TB drug MIC tested (21). Drug resistance is defined when at least 1% of growth is observed at the drug MIC when compared to growth without drug. In many cases, due to the need to obtain enough bacterial growth for DST, specimens are first grown in LJ cultures and only after growth is detected the DST is performed. This considerably delays DST results, which can take 28–42 days or longer to obtain and report.

**Table 1:** Drug susceptibility tests for implementation in mid and low-income countries with high TB burden.

	TB technology test	Method pirnciple	Cost^#^	Setting to be used	Turnaround time	Complexity	Point-of-care potential	WHO endorsed
Phenotypic DST*	BACTEC 460/960	Liquid culture	$$$	Reference lab	10-42 days	High	No	Yes
	Löwenstein-Jensen	Solid culture	$	Peripheral lab	30-45 days	Moderate	No	Yes
	7H10/7H11 agar	Solid culture	$	Peripheral lab	21-28 days	Low	No	Yes
	1G/2G Color plates	Solid culture	$	Peripheral lab	14-21 days	Low	No	No
Genotypic DST test	GeneXpert MTB/RIF	qPCR	$$$	District lab	<2h	Low	Yes, if availability of GX-Edge or Omni platforms	Yes
	GeneXpert MTB/RIF Ultra	qPCR	$$$	District lab	<2h	Low	Yes, if availability of GX-Edge or Omni platforms	Yes
	GeneXpert MTB/XDR**	qPCR	$$$	District lab	1.5h	Low	Yes, if availability of GX-Edge or Omni platforms	Yes
	TB-LAMP	Loop-mediated isothermal amplification	$$$	Peripheral lab	2h	Low	Yes	Yes
	GenoType MTBDRplus (1st line LPA)	PCR, hybridation	$$	Reference lab	5h	Moderate	No	Yes
	GenoType MTBDRs (2nd line LPA)	PCR, hybridation	$$	Reference lab	5h	Moderate	No	Yes
	FluoroType MTB and FluoroType MTBDR**	PCR, hybridation	$$	Reference lab	2.5h	Moderate	No	Yes
	Genoscholar PZA-TB**	PCR, hybridation	$$	Reference lab	1 day	Moderate		Yes
	Truenat MTB Plus	Micro RT-PCR	$$	Peripheral lab	2h	Low	Yes, onTruelab platform	Yes
	Truenat MTB-Rif Dx	Micro RT-PCR	$$	Peripheral lab	2h	Low	Yes, onTruelab platform	Yes
	Next generation sequencing (NGS)	Gene sequencing (WGS, GWAS)	$$$	Reference lab	5-10 days	High	No	
	Abbott RealTime MTB**	PCR	$$$	Reference lab	11.25h	Moderate	No	Yes
	Abbott RealTime MTB RIF/INH**	PCR	$$$	Reference lab	11.25h	Moderate	No	Yes
	Cobas MTB and cobas MTB-RIF/INH**	PCR	$$$	Reference lab	4.5h	Moderate	No	Yes

*Additional phenotypic assays include, microscopically observed drug susceptibility assay (MODS), and colorimetric redox indicator (CRI).

**Last recommendations from WHO consolidated guidelines on rapid diagnostics for TB detection, 2021.

^#^Costs are indicative using the range $-$$$ and includes set up, per test costs, and maintenance needs.

$, Low cost; $$, Medium cost; $$$, High cost.

For phenotypic DST in liquid medium, liquid medium Middlebrook (M)7H9 or M7H10 broths are normally used for mycobacterial growth indicator tube (MGIT) automated *M.tb* culture system [Becton Dickinson and Company (BD) Diagnostic Systems, Sparks, MD, USA]. A specimen is added to the MGIT in the presence of a given concentration of anti-TB drug. Bacterial growth is detected automatically by fluorescence due to oxygen consumption by the presence of *M.tb*, indicating that *M.tb* is present and it is resistant to the given drug being tested. MGIT DST is quicker than solid media, and it can take up to 14 to obtain results.

For first-line agents (INH/RIF) and some second-line anti-TB drugs [KAN, AMK, ofloxacin (OXO), levofloxacin (LEV)], phenotypic DST is generally consistent, reproducible, and widely used, except for pyrazinamide (PZA), which requires technical expertise to avoid high rates of false positive resistance using MGIT (20). Other anti-TB drugs such as fluoroquinolones [moxifloxacin (MOX), gatifloxacin], cycloserine, capreomycin, ethionamide and prothionamide, as well as re-purposed drugs clofazimine and linezolid are gaining importance to treat DR-TB, and thus, their concentrations for solid DST need to be consistently reevaluated and standardized worldwide. Further, new drugs for MDR-TB treatment such as bedaquiline and delamanid are recommended for use by WHO under specific conditions and may be added to a core MDR-TB regimen (22), and thus their phenotypic DST reevaluation will be also a priority. This is critical as although phenotypic DST is considered by many a step backwards to improve DR-TB testing, there is evidence that show that INH- and RIF resistant TB cases are missed when using genotypic DST, resulting in misleading treatments (23). This is mainly due to the fact that genotypic DST can miss the detection of novel resistance-conferring mutations that are otherwise detected by phenotypic DST (24–29).

In order to improve phenotypic DST and make it more affordable and reliable in mid and low-income countries with high TB burden rates, our group has participated in the field testing of the first generation (1G) Color Plate (1G test) (30), an inexpensive and simple diagnostic that requires minimal training (31–34). The 1G test is a non-commercial test that is based on the thin-layer agar (TLA) method with an added compound, 2,3 diphenyl-5-(2-thienyl) tetrazolium chloride (STC), that serves as an oxidation-reduction indicator that results in red *M.tb* colonies. This facilitates the detection of colonies isolated from sputum samples to diagnose active TB disease (31–34). Detection of *M.tb* using TLA has been effective for several years, is relatively inexpensive, and only requires an incubator and a 5-10X magnifier glass to confirm diagnosis (30). The 1G test can be used in rural health facilities where access to other molecular TB tests may be limited (34). This DST diagnostic test is used to identify the *M.tb* complex and detection of resistant strains to drugs such INH, RIF, and ciprofloxacin (CIP) or PZA (30, 32, 34).

**Figure 1:**
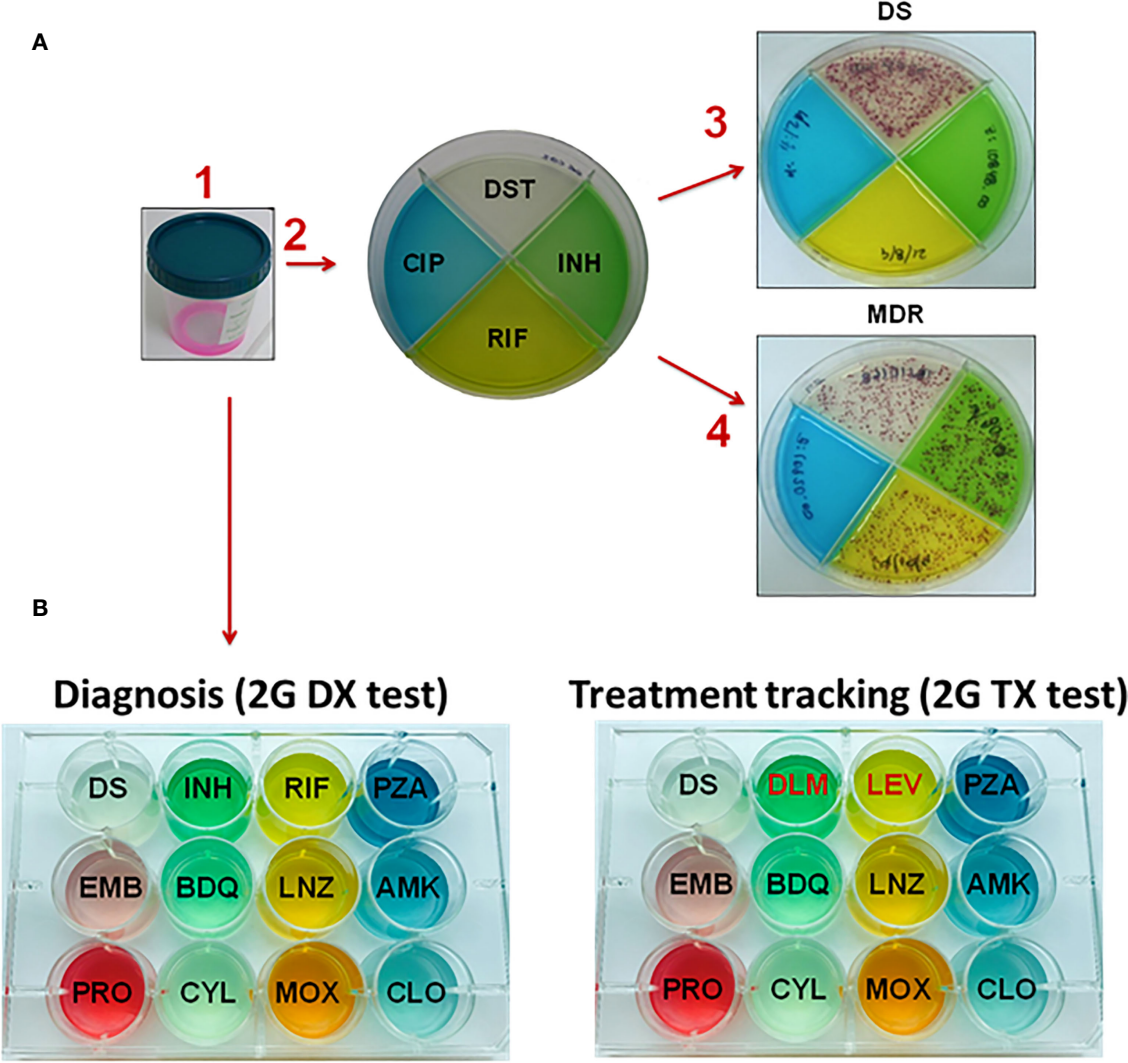
Layout of the 1G and 2G test used to diagnose drug-resistance of *M.tb* infection. **(A)** The 1G test in which four different drugs are contained in each quadrant. A patient’s sputum is collected and de-contaminated. Following the arrows, (1) sputum is mixed with decontaminant, (2) the mixture of decontaminant plus sputum (2:1, v/v) is plated into the 1G test. (3) Showing a patient with DS-TB in which colonies are only present in the DS quadrant and not in any of the quadrants with drugs present. (4) Showing a patient with MDR-TB since *M.tb* colonies grow in three of the quadrants, which include no drug (clear quadrant), isoniazid (INH, green quadrant) and rifampicin (RIF, yellow quadrant). **(B)** The 2G test showing the expansion from a quadrant plate (1G test) to 12-wells plate. In this case, the 2G test has 11 different anti-TB drugs that can be used to i) diagnose patients with TB (2G DX test), and ii) track the treatment progression to determine if a subject is responding well or not to the treatment (2G TX test). Subjects with MDR-TB and XDR-TB are by definition INH and RIF resistant; thus the treatment tracking plate has two replacement drugs, in this case DLM and LEV. Abbreviations: DS (drug susceptible); INH (isoniazid), RIF (rifampicin), PZA (pyrazinamide), EMB (ethambutol), BDQ (bedaquiline), LNZ (linezolid), AMK (amikacin), PRO (prothionamide), CYL (cycloserine), MOX (moxifloxacin), and CLO (clofazimine), DLM (delamanid), LEV (levofloxacin).

The 1G test requires minimum training, and has high sensitivity and specificity (32). Due to the STC indicator and special agar composition, *M.tb* growth is accelerated (detection in ~14 days), contaminations minimized, and colonies are visualized as red dots to the naked eye (30, 32, 34). This provides a simple concurrent readout of whether sputum samples are 1) positive for *M.tb* and 2) identification of drug resistance. The 1G test is divided into four quadrants in which one quadrant detects growth of *M.tb* (susceptible quadrant, no drug) and the other three quadrants detect drug resistance to INH, RIF, and CIP (30) (**Figure 1**). Since the 1G test has been an effective diagnostic tool for TB in rural areas such as Malawi and Ethiopia (31, 32, 34), our next step has been to expand the number of drug resistances that we can detect. Thus, we just expanded the 1G test from a quadrant culture-based test to a 12-well culture, which will provide drug resistance evaluation for 11 drugs (**Figure 1B**). This so-called 2^nd^ Generation Color Plate test (2G test) can be used to: i) diagnose primary DS- or DR-TB; and ii) monitor the treatment success of DS- and DR-TB cases. The 1G test can be used only as a diagnostic test for DS-TB and as mono-resistant to INH or RIF, MDR, or pre-XDR); however, it could not track treatment success. The purpose of the 2G test is to gauge for drug resistance and to track the progression of the treatment response. This is particularly important to provide an accurate initial diagnosis and further track and update treatment plans for patients failing their initial treatment regimen. Currently, the 2G test is under field assessment with two types of test: an initial diagnostic test (2G DX), and a treatment progression monitoring test (2G TX) designed to monitor DR-TB patient treatment evolution. Drug composition for both 2G DX and 2G TX is depicted in **Figure 1B**, including the latest anti-TB drugs to treat DR-TB.

### Genotypic DST

Genotypic DST detects specific DNA mutations associated with resistance to specific anti-TB drugs in the *M.tb* genome. Genotypic DST has many advantages, including fast results, testing standardization and high throughput. However, the need of funding to support expensive equipment and supplies, a maintenance plan and quality assurance, specialized labs, and an uninterrupted electrical supply, as well as, an excessive cost per test, holds genotypic DST for being implemented globally for the management of DR-TB, especially in mid- and low-income regions with high TB incidence.

The development and implementation of nucleic acid amplification tests (NAATs) have revolutionized the TB and RR/MDR-TB field. As NAATs detect *M.tb* DNA in specimens, their performance correlates directly with the quality of the specimen tested. In the last update for rapid diagnostics for TB detection, WHO endorses three NAAT classes for genotypic DST: low, moderate and high based on the type of technology (automated or hybridization), target conditions (DS-, DR-TB), and complexity for its implementation (equipment, training, infrastructure) (18). Examples of low complexity tests include the Xpert MTB/RIF Ultra or Xpert XDR (Cepheid, Sunnyvale, CA, USA). Moderate complexity automated tests include the Abbott RealTi*m*e MTB and RealTi*m*e MTB RIF/INH (by Abbott, Abbott Park, Illinois, USA), FluoroType MTBDR and FluoroType MTB (by Hain Lifescience, Nehren, Germany), BD MAX™ MDR-TB (by BD, Fraklin Lakes, New Jersey, USA), cobas MTB and cobas MTB-RIF/INH (by Roche, Basel, Switzerland) among others. High complexity tests include hybridization based tests such as the Genoscholar PZA-TB (Nipro Corporation, Tokyo, Japan) (18).

Current molecular testing of the first line drugs INH and/or RIF includes the Xpert and commercial line probe assays (LPAs), such as the Nipro NTM + MDRTB detection kit 2 (Nipro Corporation, Tokyo, Japan) and the MTBDRplus assay (Hain Lifescience, Nehren, Germany). The Xpert assay targets the detection of *M.tb* as well as detection of those mutations that confer RIF resistance. As this drug is rarely resistant by itself, RIF resistance (mutations in the *rpoB* gene) is used as a surrogate to define MDR-TB. The detection of RR *via* Xpert compares well with phenotypic DST methods (35). An advantage of LPAs is that these detect both INH and RIF associated mutations. INH resistance conferring mutations are defined in *inhA* (giving low resistance) and *KatG* (giving high resistance) and these account for ~90% of INH resistance as detected using phenotypic DST (36–39). The other ~10% of INH resistance is related to undetermined mutations in the *M.tb* genome, although mutations on *inhA* promotor and coding regions are reported to drive this resistance (40–43).

As a genotypic DST, in settings that can afford it, Xpert is used for initial diagnostic test for all people presented with TB compatible symptoms, followed by, in some cases, phenotypic DST such as culture. Xpert can be performed using both pulmonary and extrapulmonary specimens. Further, the Xpert Edge is now deployed with the goal of being more user friendly, as well as reducing testing cost and enhance efficiency, with a battery support to reach rural areas in need. Indeed, the Xpert XDR-TB is in development to fit the GeneXpert and GeneXpert Edge platforms to simultaneously detect mutations related to INH, fluoroquinolones, and 2^nd^ line anti-TB agents (18, 44, 45). However, future studies will need to focus on the mechanism(s) of resistance behind the new generation of drugs developed or repurposed such as bedaquiline, delamanid, pretomanid, and linezolid among others, in order to develop novel molecular tests to capture *M.tb* developed resistance to these drugs. Nonetheless, the costs of Xpert technology is prohibited for many high TB burden countries, were cartridge supplies cost depend on owning or leasing the instrument, varying from ~$10 (own instrument) to $100 (for leased instruments, with variations depending on the country) (46, 47), making many health centers to dismiss its use. Further, patients with previous TB may have residual DNA in sputum making Xpert prone to false–positive results (28, 48) that together with its suboptimal sensitivity in special populations such as children, people living with HIV (PLWH) and extrapulmonary TB patients (49, 50) has undermined its impact in long-term patient outcomes (51, 52). However, this technology is evolving and the possibility of generating a multi-array to diagnose co-infections may make the Xpert more cost-effective in the future. This is already thought for dual TB/COVID-19 testing, especially after the deployment of the Xpert Xpress SARS-CoV-2, which can also minimize the impact of using current Xpert equipment to diagnose only COVID-19 in detriment of TB (44).

The Truenat MTB tests (MTB, MTB plus and MTB-RIF Dx) developed by Molvio diagnostics Pvt. Ltd (Bangalore, India) are chip-based real time micro PCR detection assays for diagnosing TB. These tests can be deployed at peripheral health care settings with minimal infrastructure and depend on battery operated devices that extract, detect and amplify DNA from patient sputum samples for detection of DS- and RR-TB. WHO recommends these assays to be used as a diagnostic test for TB rather than smear microscopy/culture (53). An ongoing multicenter prospective clinical evaluation study in 19 clinical sites, shows good diagnostic accuracy results, despite some evidence of imprecision and inconsistency for sensitivity results and the need of more studies to evaluate the affordability and cost-effectiveness of its implementation (53, 54).

First line LPAs (FL-LPAs) for detection of INH/RIF resistance, as well as second-line LPAs (SL-LPAs) for the detection of resistance to second-line injectable drugs (mutations in the *rrs* and *eis* promoter genes) and fluoroquinolones (mutations in the *gyrA* and *gyrB* genes) are also commercially available (18). WHO recommends the use of commercial LPAs as a rule-out test for XDR-TB. However, caution is recommended because the detection of mutations in the *rrs* and/or eis genes by SL-LPA does not mean that the tested *M.tb* strain is resistant to all the drugs within the second line injectable drug class. Further, although a positive LPA result is reliable in smear positive sputum samples, its diagnostic performance in smear negative sputum samples is low, and thus, adopting LPA does not eliminate the need for phenotypic DST capacity, especially in regions with high DR-TB incidence (18, 55, 56). Another limitation for LPA wide implementation is the need of high-standard molecular facilities to perform the test, which makes its implementation highly challenging in many mid- and low income high TB burden regions (18, 44).

Like the Xpert and LPA, other NAATs are the Gen-Probe MTD test (57) and Pyrosequencing (PSQ) (58). Although there is a push for NAATs becoming a standard diagnosis practice for all presumptive TB cases (59), WHO recommends that NAAT must not replace phenotypic DST such as culture or MGIT. A positive NAAT result is recommended to be supported by other tests such as culture, as well as a negative NAAT result should not be used to definitely exclude TB. Overall, increased use of NAAT in DR-TB diagnosis could decrease the time-to-treatment initiation; however, differential implementation, cost and access to NAAT is a limitation in high TB burden communities.

Although still far away from its routine implementation in the field, next generation sequencing technologies (NGS) (60), such as whole-genome sequencing (WGS) of *M.tb* followed by association with phenotypic resistance using genome-wide association studies (GWAS) are also promising tools for the diagnosis of DR-TB (61, 62). Indeed, GWAS has been used for both, identification of host and *M.tb* traits implicated in the evolution of DR-TB. Thus, host GWAS studies have identified polymorphisms correlated to the heritability of host susceptibility to TB (63), as well as, *M.tb* GWAS studies revealed new resistance genes and regulatory regions implicated in the *M.tb* resistance to 11-anti-TB drugs (64). Some of the advantages of WGS-based diagnostic strategies (65) is the accuracy to diagnose DR-TB (66); however, due to much higher costs compared to traditional microbiological techniques and the need for computing and bioinformatics capabilities, NGS in resource-constrained settings is still far from being implemented (7).

Currently, several novel molecular tests for TB detection are under development at different levels of care: from reference labs to decentralized health care settings to be used as POC tests (18). Although not WHO endorsed yet, the Xpert GX-Edge and Omni, WGS, POC NAATS, and centralized DST can add to the current WHO endorsed LPA, Xpert, TB-LAMP, and Truenat to diagnose DS- and DR-TB, although caution needs to be considered for some of these tests as there is very low certainty of evidence for test accuracy for some of them in determining DR-TB (e.g. Truenat by Molbio) (67–69). For detailed current molecular diagnostic tests in the pipeline (e.g. POC NAATs such as Q-POC from QuantuMDx, indigenous Chinese diagnostics, high-throughput centralized diagnostic test, and next generation sequencing) see the review by MacLean *et al.* elsewhere (44).

## TB Immunodiagnostic Tests

### Immunodiagnostics of Latent *M.tb* Infection (LTBI)

A challenge to TB control worldwide is being able to identify the individuals who are going to progress to develop active TB disease upon infection. Targeting these individuals for latent *M.tb* infection (LTBI) treatment can substantially reduce active TB risk. Thus, testing for LTBI is indicated when the risk of developing disease is increased, such as recent contacts of new TB patients, or anyone with a potential exposure that has compromised capacity to contain the infection due to altered immunity (70). These include PLWH, poorly-controlled diabetes, taking immunosuppressive medications (e.g. steroids), young (infants and children) or old age, and other conditions including malnutrition (71, 72). Thus, it is important to identify these individuals, but there is no diagnostic gold standard for LTBI, and particularly for those with high risk of developing active TB.

Two types of LTBI screening tests are available: the tuberculin skin test (TST) and interferon gamma (IFN-γ) release assays (IGRAs). They represent indirect markers of LTBI based on immunological memory of T lymphocytes to *M.tb* antigens. These tests have an acceptable performance and are widely used in the clinical setting, but have limitations: Neither has a gold standard for confirmation of LTBI, they cannot distinguish LTBI from active TB, nor the various stages of the spectrum of LTBI, and they have a low positive predictive value (about 2-3%) for progression from LTBI to active TB disease (73).

The TST consists of the intradermal injection of mycobacterial purified protein derivative (PPD), with recall immunity inducing a delayed-type hypersensitivity measured by local induration approximately 48 h post-administration (74). Limitations include false positives due to cross-reactivity with non-tuberculous mycobacteria (NTM) and possibly with BCG-vaccinated individuals, particularly if re-vaccinated after infancy and multiple times (72). Specificity is 97% in regions where BCG vaccination is not used, and lowers to about 60% in regions where BCG is applied (75, 76). False negatives occur in patient groups who are immunosuppressed (e.g. HIV, malnutrition) or the elderly due to reduced intradermal immunity (72, 77).

IGRA tests are based on the detection IFN-γ production by peripheral blood lymphocytes in response to specific peptides from *M.tb*, mostly early secretory antigenic target protein (ESAT)-6 and culture filtrate protein (CFP)-10. These peptides are more specific than PPD antigen because they are not encoded in the genomes of most other mycobacteria, with assays reaching >95% specificity. IGRAs are conducted ex-vivo after a blood draw, and hence, only require one office visit. There are two commercial IGRA: The T-SPOT.*TB^®^* (Oxford Immunotec Ltd, Abingdon, United Kingdom) and the QuantiFERON^®^, which has had several versions, with the current being the QuantiFERON-Gold Plus (QFT-Plus; Qiagen, Germantown, MD, USA) (74). The T-SPOT.*TB* is an enzyme-linked immunosorbent spot assay, while the QuantiFERON uses an ELISA format. The sensitivity of the T-SPOT.*TB* is generally higher compared to QuantiFERON or TSTs (approximately 90%, 80% and 80%, respectively), but this will vary between study populations. Further, although IGRA is a useful diagnostic method for differentiating TB from NTM diseases, in China for example (78), IGRA shows limited value in this discrimination (79). Further, IGRA does not differentiate *M.tb* from *M. kansassii*, *M. szulgai*, and *M. marinum* because these mycobacterial species also have ESAT-6 and CFP-10 (78, 80). Among the elderly both IGRAs perform similarly, which contrast with the reduced sensitivity of TST in this population (77).

In recent years, the focus has shifted to improving the specificity of immunodiagnostics to identify active TB disease *vs*. LTBI. Certain studies have investigated the diagnosis of active TB using multiplex cytokine and chemokine analysis to improve the sensitivity of IGRAs (81–83). Alternatively, the focus is on depicting *M.tb*-specific T-cell responses *via* flow cytometry measuring both phenotype and function of the T cells (84). Other options used the QuantiFERON-TB Gold In-Tube (QFT-GIT) supernatant in people with and without HIV co-infection, to increase the specificity for active TB diagnosis *vs.* LTBI by looking at differences in multiple host cytokine and chemokine biomarkers assessing multiple unstimulated cytokine/chemokines (IFN-γ, MIP-1β, and TGF-α) coupled with stimulated cytokines (TGF-α and VEGF) (84, 85).

Assessing the activation of *M.tb*-specific T cells is an active field to develop TB immunodiagnosis, for example looking at CD4^+^ IFN-γ^+^ T cells for HLA-DR, CD38, and Ki-67 markers, which shows 100% sensitivity and 95% specificity for active TB (86–88). Phenotypic changes on *M.tb*-specific CD4 T cells are also used as surrogate markers for TB treatment efficacy and can help to discriminate between TB (profile: CD38^pos^, CD27^low^), treated TB (CD38^neg^, CD27^low^), and LTBI (CD38^neg^, CD27^high^) (89). Other studies have looked at *M.tb*-specific TNF producing CD4^+^ T cells along with the detection of *M.tb*-specific CD8^+^ T cells and found that, in combination, these immunological assays have a 81.1% sensitivity and 86.5% specificity in the diagnosis of active TB (90–92). Active TB is also significantly associated with an increase in CD27- *M.tb*-specific CD4+ T cells, where evaluating CD27+ CD45RA- CD4+ IFN-γ^+^ T cells increases diagnostic accuracy of active TB *vs.* cured TB or LTBI even further (93). Indeed, the combination of different blood biomarkers – namely, CD27, CD45RA, and TNF – within the population of CD4^+^ IFN-γ^+^ T cells provides a good acceptable diagnostic accuracy regarding active TB *vs.* LTBI with 92% sensitivity and 97% specificity while reducing the need to obtain a sputum sample (93). Notably, however, these improvements in immunodiagnostics are not POC and do not allow for the detection of DS- *vs.* DR-TB cases within a population of individuals that have active TB, regardless of their HIV status. As aforementioned, the field is still lacking in POCs to identify DR-TB and provide information about the drug resistance profile, particularly in high TB burden areas where DR-TB is more common.

Interestingly, there are studies in progress to determine when a LTBI case will progress to active TB. An expert consultation was convened by WHO in 2015 to develop target product profiles (TPPs) and an evaluation framework for tests aiming to predict progression from LTBI to active TB (94). In this context, indirect blood PCR-based biomarker tests looking at the expression of host immune response genes to *M.tb* infection are being developed and validated as diagnostic tools that can also predict progression from LTBI to active TB, targeting all forms of TB and patient populations (95). Supporting this, a systematic comparison study of 16 host-derived gene expression signatures (96) found that 7 out of 16 signatures predicted progression from LTBI to active TB disease 6 months prior to sputum conversion (e.g. incipient TB), indicating that some host-response-based diagnostics could be generalizable across diverse patient populations and thus, considered for clinical implementation (97).

### Immunodiagnostics of Active TB

One of the key pillars of the End TB strategy is based on early TB diagnosis (17, 98). In this regard, some time ago, a report generated together by WHO and the Foundation for Innovative New Diagnostics (FIND) identified four major TPPs, defining the targets and specifications that new diagnostic TB tests should meet (1): a POC non-sputum test capable of detecting all forms of TB (immunodiagnosis biomarker test); (2) a simple, low-cost POC test performed in clinical/rural settings (e.g. healthcare post) to screen and identify those who need further TB testing (triage test, immunodiagnosis or direct Ag-detection); (3) a POC sputum test to detect pulmonary TB to replace the widely used smear microscopy (the smear-replacement test); and (4) a rapid and efficient DST that can identify those in need of first-line drug treatment (a rapid DST test) (99, 100)

Despite the number of TB diagnostics available, there are still challenges to deploy these tests to urban and rural TB healthcare post and facilities (30, 101). Important parameters to consider in the development of POC diagnostics are, an easily accessible sample, rapid results, high sensitivity and specificity, and cost (less than USD $4 in the location where the test is performed) (101). Currently, available POC diagnostics include the Determine™ lipoarabinomannan (TB-LAM) Ag test (antigen detection), FujiLAM TB test (antigen detection), LIODetect^®^ TB-ST TB Rapid Test developed by LIONEX (antibody detection), and smartphone diagnostics discussed below.

Several POC TB diagnostics are based on the detection of a unique cell envelope component of *M.tb* complex, the mannose-capped lipoarabinomannan (ManLAM) (102). ManLAM is a lipoglycan antigen (Ag) present with a defined role in the survival of *M.tb* during infection (102–104). Specifically, the Determine™ TB-LAM Ag test (Abbott Rapid diagnostics, Abbott Park, IL USA) is a rapid lateral flow Ag-detection POC diagnostic TB test that uses non-invasive samples such as urine to detect undisclosed motifs on the *M.tb* ManLAM structure (104). This diagnostic test is considered to be a ‘real’ POC test, as it can be used and result read in the field within 25 minutes. This test can be useful to confirm active TB cases, specifically in PLWH, or with sputum smear negative results; however, the test has low sensitivity (52% sensitivity and 98% specificity) (104, 105). To improve upon the sensitivity of this test, our lab demonstrated a simple biochemical step using α-mannosidase to treat the urine samples to cleave the mannose caps on the structure of LAM, providing an increased affinity for LAM antibodies to bind and recognize the epitopes (105). This additional step only added 15 minutes and $0.50 to the cost (final $3.50 per test). The FujiLAM TB test is another novel POC that detects LAM in the urine of patients with clinical symptoms of active TB disease (106). Specifically, the FujiLAM test detects the 5-methyl-thio-*D*-xylofuranose (MTX) motif found on the non-reducing terminal end of the ManLAM structure. The MTX motif reduces cross-reactivity with *M.tb* complex, thus it could be considered a rapid POC TB test that detects positive active TB cases (107, 108). The FujiLAM test has shown a superior diagnostic sensitivity in inpatients with HIV compared to the Determine™ TB-LAM Ag test (70.4% *vs.* 42.3%) (109). The FujiLAM test also shows similar sensitivities (at 75%) in both PLWH and people without HIV infection (110). Furthermore, FujiLAM test shows a substantial higher sensitivity compared to the Determine™ TB-LAM Ag test for detecting extra-pulmonary TB in PLWH (111). Although both the Determine™ TB-LAM Ag and FujiLAM TB tests are rapid POC diagnostics, there is still uncertainty of their sensitivity among HIV negative patients and cannot differentiate between DS- *vs.* DR-TB (112).

Additionally, TB serological tests are used in mid and low income countries to detect the presence of antibodies for TB. One such serological test is the Anda-TB test developed by Anda Biologicals, which looks for the presence of either IgG, IgA, or IgM antibodies specific for the *M.tb* A60 antigen. Meta-analysis of the Anda-TB IgG test revealed a pooled sensitivity of 76% and specificity of 92% in individulas that were acid-fast bacilli (AFB) smear positive, and 59% sensitivity and 91% specificity in AFB smear negative individuals (113). Another TB POC is the LIODetect^®^ TB-ST Rapid Test developed by LIONEX, which detects IgG, IgA and IgM antibodies to *M.tb* antigens in serum, plasma, or whole blood under 20 minutes with 65% sensitivity and 98% specificity (114).

Another aspect of POC diagnostics is the addition of simple adaptors to smartphones for reading results, simplifying the interphase required for TB diagnostics and improving accessibility given their use in daily life, high connectivity and functionality while being portable (115). Smartphones can be equipped with simple adaptors and apps that can be used to capture images for visualization and TB diagnosis (116). Another incentive for smartphones being added to the POC arsenal is that there are project initiatives such as Fair Phone and ARA that can provide low cost manufacturing on a large scale (115). Thus, new technologies and research efforts are imperative, especially in the context of POCs, to provide reliable results in a timely manner and at a low cost (100, 101).

On the light of immunodiagnosis specifically for DR-TB, nothing is developed or is known to be in the discovery pipeline. In this regard, metabolic studies in specimens isolated from DS- and DR-TB patients may bring some light into the discovery of biomarkers that are solely expressed when a person is infected with DR-*M.tb*. However, this seems not plausible. It will be challenging to identify a host biomarker panel to differentiate the drug resistant profile of the infecting *M.tb* strain. Even, if it is doable, a push back from the TB scientific community is expected, as evidences exists that immunodiagnostic tests can be inaccurate and thus, do not improve patient outcomes, and are considered suboptimal tests to be used for pulmonary and extrapulmonary active TB diagnosis in mid and low income countries with high TB burden.

## Conclusion

Despite recent and encouraging advances towards TB elimination, the ongoing COVID-19 pandemic disruption has undermined these achievements. Cases of *M.tb* infection reactivation in people recovered from severe COVID-19 are reported (117, 118). The struggle of using recourses for TB diagnosis and care to manage the current demand on COVID-19 is expected to have an impact on TB control (119–121). One of the key aspects to target TB elimination is early TB diagnosis that needs affordable and high-sensitivity POC tests able to diagnose pulmonary and extra pulmonary TB in adults, children, and PLWH (100). To fill current gaps in DS- and DR-TB diagnosis, it is also imperative to develop novel sputum and non-sputum based POC or semi-POC (deployable in rural areas and health-post with minimal resources) diagnostic tests able to characterize drug resistance to improve proper TB care and long term TB treatment outcomes. We will need to consider revisiting phenotypic DST, as genotypic DST, despite its many advantages, has many social-economic barriers, most importantly the high cost that mid and low-income countries with high TB burden cannot afford, even with the assistance of WHO and the good faith of companies developing these technologies. In this context, even with WHO endorsement, national policy needs to be established to ensure test adoption, scale up, and implementation (122). At the end, the cost of supplies, the need of sophisticated technology requiring repeatedly calibrations and staff training; plus, the fact that current genotypic DST cannot identify resistance to current drugs (such as bedaquiline, delamanid and others), and misses the detection of some unknown mutations driving resistance to first line drugs, makes an improved phenotypic DST a need to cover a current global public health demand.

## Limitations

This narrative review is not a systematic review of the literature, and therefore it might unintentionally not include all published papers related to the topic discussed.

## Author Contributions

All authors listed have made a substantial, direct, and intellectual contribution to the work, and approved it for publication.

## Funding

This study was partially supported by the National Institutes of Health (NIH)/National Institute of Allergy and Infectious Diseases (NIAID) AI150445 to JT.

## Conflict of Interest

The authors declare that the research was conducted in the absence of any commercial or financial relationships that could be construed as a potential conflict of interest.

## Publisher’s Note

All claims expressed in this article are solely those of the authors and do not necessarily represent those of their affiliated organizations, or those of the publisher, the editors and the reviewers. Any product that may be evaluated in this article, or claim that may be made by its manufacturer, is not guaranteed or endorsed by the publisher.
